# Recent Advances in Nanotechnology-Based Targeted Delivery Systems of Active Constituents in Natural Medicines for Cancer Treatment

**DOI:** 10.3390/molecules28237767

**Published:** 2023-11-24

**Authors:** Yu Hu, Jizheng Song, Anjie Feng, Jieyu Li, Mengqi Li, Yu Shi, Wenxiu Sun, Lingjun Li

**Affiliations:** School of Pharmacy, Shandong University of Traditional Chinese Medicine (TCM), Jinan 250355, China; huyu01230304@163.com (Y.H.); songjizheng345@163.com (J.S.); faj1130439892@163.com (A.F.); 15069001951@163.com (J.L.); limengqi7475@163.com (M.L.); shiyu2021214@163.com (Y.S.); sunwenxiuxiu1022@163.com (W.S.)

**Keywords:** nanoparticles, tumor targeting, natural medicines

## Abstract

Owing to high efficacy and safety, natural medicines have found their way into the field of cancer therapy over the past few decades. However, the effective ingredients of natural medicines have shortcomings of poor solubility and low bioavailability. Nanoparticles can not only solve the problems above but also have outstanding targeting ability. Targeting preparations can be classified into three levels, which are target tissues, cells, and organelles. On the premise of clarifying the therapeutic purpose of drugs, one or more targeting methods can be selected to achieve more accurate drug delivery and consequently to improve the anti-tumor effects of drugs and reduce toxicity and side effects. The aim of this review is to summarize the research status of natural medicines’ nano-preparations in tumor-targeting therapies to provide some references for further accurate and effective cancer treatments.

## 1. Introduction

According to global cancer statistics in 2020, the number of newly diagnosed cancer cases in 2020 was 19.3 million, and nearly 10.0 million people died from cancer. Lung cancer and breast cancer were the most common cancers in males and females, respectively, and were also the main causes of cancer death. As the report also noted, the incidence and mortality rate of cancer would continue to rise [1]. In light of this, an effective and safe therapeutic approach for cancer is urgently needed.

The ultimate objective of cancer treatment is to eliminate tumors while inflicting no damage on normal tissues. The conventional cancer therapies in clinical practice mainly comprise surgery, chemotherapy, and radiation therapy [2]. However, the early stages of cancers are generally asymptomatic, and most cancers are already in the advanced stage when diagnosed [3,4]. One of the intractabilities that has appeared in studies is how to treat advanced and malignant tumors because surgical treatment cannot completely remove deep tumors, which may subsequently lead to cancer recurrence and metastasis [5]. Given the circumstances, the majority of patients with advanced cancers tend to be treated with chemotherapy [6,7]. The commonly used chemotherapy drugs in clinic include doxorubicin, gemcitabine, cisplatin, 5-fluorouracil, etc., which are usually used alone or in combination to inhibit tumor growth [8,9]. Nevertheless, conventional chemotherapy treatments still leave much to be desired, which suffer from many problems, including non-specific toxicity caused by insufficient targeting and multi-drug resistance (MDR) [10,11]. Driven by the current needs of clinical medicine, it is of great necessity to develop new agents with specificity and selectivity to provide improved therapeutic strategies.

**Figure 1:**
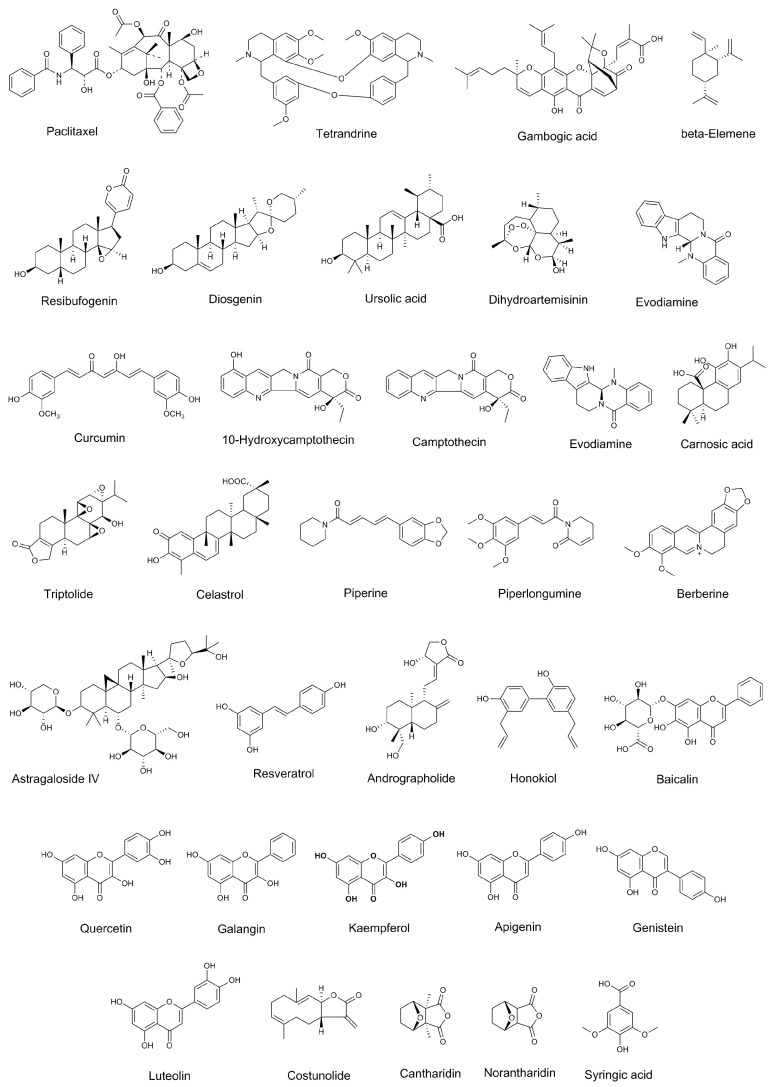
Chemical structures of compounds with antitumor activities in natural medicines.

With the gradual deepening of research on anti-tumor mechanisms, natural medicines, a rich source of potent anticancer agents, have attracted increasing attention worldwide. Compared with conventional chemotherapy drugs, natural medicines have the characteristics of multi-component, multi-pathway, and multi-target actions and play a significant role in the prevention and treatment of cancer. For example, curcumin (CUR) and resveratrol (RES) can not only directly play a cytotoxic role on tumor cells but also treat cancer by regulating the tumor microenvironment [12,13,14]. Moreover, the combination of natural medicines with chemotherapy drugs to treat cancer can overcome or reverse MDR, improve efficacy, and reduce the toxic side effects, becoming a new strategy for tumor treatment in recent years [15,16]. Chemical structures of compounds with antitumor activity in natural medicines are shown in Figure 1. 

**Figure 2:**
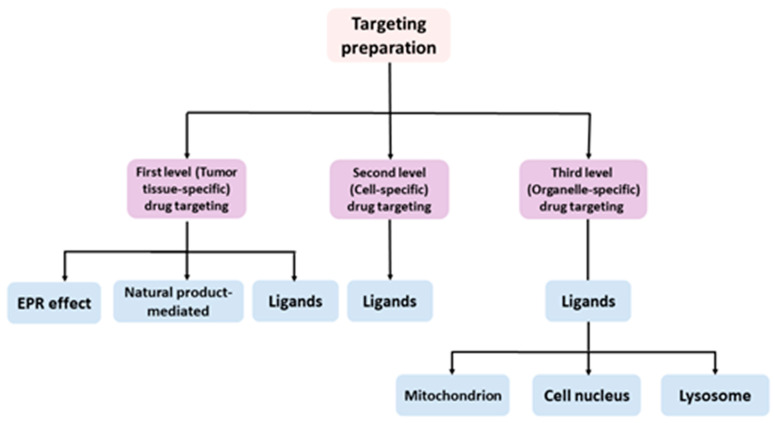
Classification and targeting methods of targeting preparation.

**Figure 3:**
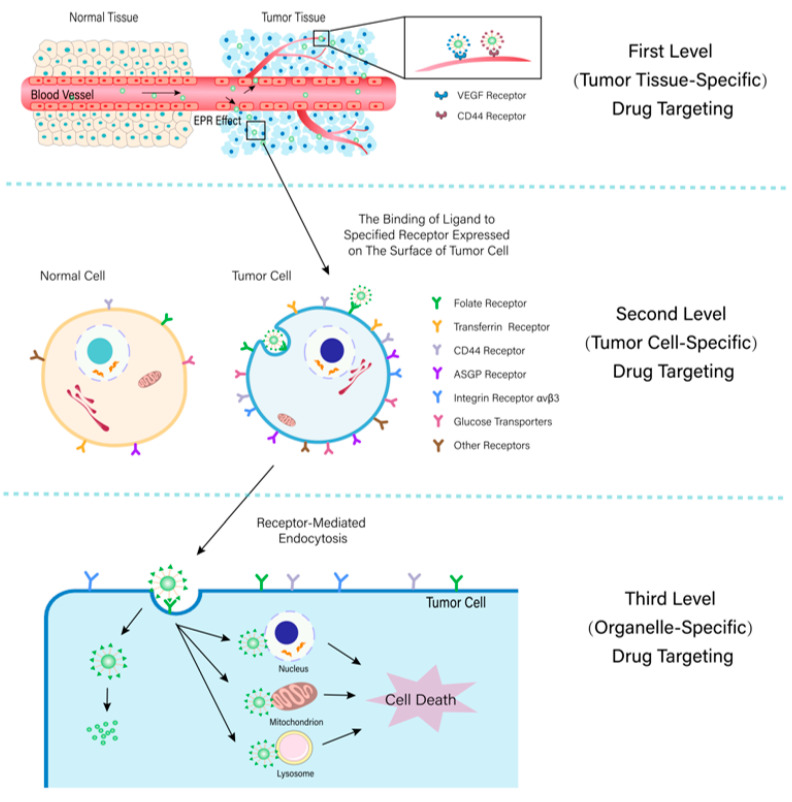
The mode of action of tumor tissue-specific, cell-specific, and organelle-specific targeting.

However, the majority of effective ingredients in natural medicines have the deficiencies of low solubility, low bioavailability, and poor tumor targeting efficiency, which limit their clinical applications [13,17]. Using nano-targeted formulations as drug delivery systems can not only solve the problems mentioned above but also achieve simultaneous deliveries of multiple drug components [18]. According to the different targeting locations, targeting preparations can be classified into first-level targeting, second-level targeting, and third-level targeting, which deliver drugs to tumor tissues, cells, and organelle, respectively (The classification and targeting methods of targeting preparation are shown in Figure 2. The mode of action of tumor tissue-specific, cell-specific and organelle-specific targeting are shown in Figure 3) [19,20,21]. Under the guidance of action mechanisms or clinical treatment needs, one or more targeting methods can be selected to deliver drugs to specific tumor tissues, cells, or organelle in order to increase the concentrations of drugs in the tumor site, improve the anti-tumor effects of drugs, and reduce the toxic side effects on normal tissues and organs.

In summary, the combination of natural medicines with nano-targeted technologies to form a new type of natural medicines delivery system for tumor treatment is a promising research direction. The aim of this review is to summarize the research status of natural medicines nano-preparations in tumor-targeting therapy to provide some references for further accurate and effective cancer treatment.

## 2. First-Level (Tumor Tissue-Specific) Drug Targeting

### 2.1. EPR Effect-Mediated Drug Targeting

Owing to the high permeability of tumor blood vessels, nano-preparation with particles of a size less than 200 nm can enter the tumor stroma and be retained by impaired the lymphatic system. This phenomenon is known as the high permeability and retention effect of solid tumors (EPR effect) [22]. It is generally believed that the delivery system mediated by the EPR effect can effectively deliver nano-carriers to tumor tissues through passive transport [23,24]. In recent years, a series of studies on nano-preparation have been carried out at home and abroad, including micelle, liposome, nanoemulsion, and other dosage forms.

#### 2.1.1. Micelle

When surfactant concentrations exceed the critical micelle concentrations (CMCs), soluble surfactants start to attract and associate with each other together to form micelles in an aqueous solution [25]. It has been repeatedly shown that the encapsulation of hydrophobic bioactive compounds inside this carrier system can improve their water solubility and bioavailability. According to the molecular weight of surfactants, micelles can be divided into low molecular micelles and polymer micelles.

As traditional excipients in the pharmaceutical field, low molecular surfactants are also good candidates for the preparation of micelles. In the early stage, phospholipids (amphoteric surfactants) and bile salts (anionic surfactants) were commonly used to prepare micelles, which could increase the solubility and anti-tumor efficacy of drugs [26,27,28]. Taking bile salts and phosphatidylcholine as carrier materials, Jiao et al. [26] prepared ISA (andrographolide derivative)-loaded mixed micelles with an encapsulation efficiency of 86.34%, a drug-loading rate of 4.87%, and an average particle size of 148.3 nm. The pharmacokinetic experiment displayed that compared with free drugs, the area under the curve (AUC_0–12 h_), in vivo retention time (MRT), and elimination half-life (t_1/2_) of micelles had increased by 2.62, 1.47, and 1.40 times, respectively, indicating that micelles could effectively improve the blood circulation time and bioavailability of ISA.

Despite all of these advantages, the application of micelles prepared from bile salts is frequently hampered because the alkaline micellar system is unsuitable for drugs that are unstable in alkaline environments. Non-ionic surfactants, which have been applied in establishing drug-carrying micelles, are a promising alternative carrier material for bile salts and can overcome the aforementioned drawback. Liang et al. [29] designed a paclitaxel (PTX)-loaded phospholipid-Tween-80 mixed micelle that exhibited stronger cytotoxic activity to cervical cancer cells HeLa and lung cancer cells A549 than free PTX (*p* < 0.01). The mechanism of action might be that Tween-80 could disrupt fatty molecules and bilayer membranes, evidently enhancing the permeability of cell membranes to PTX. An in vivo pharmacokinetic experiment demonstrated that the mixed micelle possessed higher bioavailability, with AUC_0–t_ increasing by 1.3 times compared to the free PTX.

However, low molecular micelles have many inadequacies, including inflexibility in design and restricted cytotoxic effects on cancer cells and, which are more important, low molecular surfactants such as Tween-80 are the main anaphylactoid constituents of natural medicine injections. These factors have limited their clinical applications [30,31]. Therefore, in recent years, researchers have mostly switched to using amphiphilic polymers with low toxicities, excellent biodegradability, and satisfactory biocompatibility as carrier materials for the preparation of drug-carrying micelles.

Polymeric micelles, a disperse system with core–shell structure, are formed by a self-assembly of amphiphilic block copolymers in an aqueous solution. The average particle size of polymeric micelles ranges from 20 to 200 nm [32,33]. Amphiphilic copolymer carrier materials, including diblock, triblock, or pentablock copolymers (AB, ABA, ABC, or ABCBA block copolymers) [32,34,35,36], are commonly synthesized from two or more hydrophilic and hydrophobic copolymers through the esterification reaction, ring opening polymerization, or other methods [13,37]. The normally used raw materials can be divided into three categories: (1) hydrophilic polymers, such as polyethylene glycol (PEG), poly (vinyl pyrrolidone), poly(2-vinylpyridine), etc. [36,38,39]; (2) hydrophobic polymers, such as poly lactic-co-glycolic acid (PLGA), polycaprolactone (PCL), polylactic acid (PLA), etc. [36,40,41,42]; (3) amphiphilic block copolymers, mainly including D-alpha-tocopheryl polyethylene glycol succinate (TPGS), Soluplus^®^, and Pluronic^®^ (F127, F68, and P123) [13,43,44,45,46,47,48,49]. The classification and structures of the aforementioned polymers are shown in Table 1. During the micellization process, hydrophobic chain segments aggregate internally to form an inner core, serving as a reservoir for poorly water-soluble drugs. The hydrophilic outer corona is mainly composed of hydrophilic segments that can refrain from the clearance effect of the endothelial network system and prevent micellar particles from aggregating [25]. Such a core–shell structure not only enables the polymer to be well dispersed in aqueous solution but also provides a sufficient hydrophobic microenvironment for insoluble drugs due to its large relative molecular weight [45]. Therefore, compared with low molecular micelles, the encapsulation efficiency, drug loading, stability, anti-tumor effect, and bioavailability of polymer micelles are significantly improved [50]. Andrographolide (ADG) isolated from *Andrographis paniculata* (Burm. f.) Nees has anti-cancer and anti-inflammatory activities, but high hydrophobicity and poor bioavailability limit its clinical application [51]. Therefore, researchers prepared PLGA-PEG-PLGA/ADG polymer micelles with encapsulation efficiency and drug loading of 92% and 8.4%, respectively. The particle size was 124.3 ± 6.4 nm and could remain stable even after 15 days stored at 4 °C. ADG-loaded micelles had more outstanding anti-tumor ability and higher bioavailability compared to free ADG. In vitro tests proved that after 48 h of treatment, ADG micelles induced stronger cytotoxicity on breast cancer cell lines MAD-MB-231 than free drugs, with IC50 values of 7.45 ± 1.21 and 19.4 ± 2.52 µM, respectively. This might be related to the effect of inhibiting G2/M phase cell cycle and promoting cell apoptosis. In vivo experiments demonstrated that ADG micelles could continuously release within 48 h, and AUC_0–∞_ and MRT increased by 2.7- and 2.5-fold, respectively, compared to the original drug [52].

**Table 1:** The classification and structures of the common polymers used for forming micelles.

Classifications	Polymers	Structures	Ref.
Hydrophilic polymers	Polyethylene glycol (PEG)		[34,36]
	Poly (vinyl pyrrolidone)	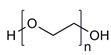	[39]
	Poly(2-vinylpyridine)	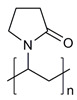	[38]
Hydrophobic polymers	Poly lactic-co-glycolic acid (PLGA)	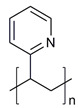	[35,41]
	Polycaprolactone (PCL)	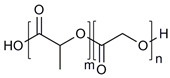	[42,53]
	Polylactic acid (PLA)	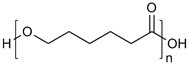	[34,40]
Amphiphilic block copolymers	D-alpha-tocopheryl polyethylene glycol succinate (TPGS)	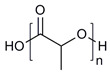	[43,44]
	Soluplus^®^	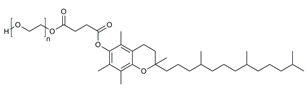	[47]
	Pluronic^®^ (F127, F68, P123)	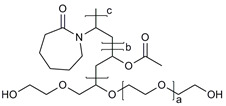	[45,46,49,54]

Polymer-mixed micelles (PMMs), formed by two or more different types of copolymers, can eliminate complex synthesis schemes of carrier materials, improve the stability of nano-micelles, enhance the compatibilization of hydrophobic compounds, and increase the anti-tumor efficacy of drugs [47,55]. In addition, PMMs have a smaller particle size (usually less than 100 nm), which is more conducive to cellular uptake and is a promising drug delivery system [55,56].

It is widely acknowledged that TPGS, which possesses inhibitory effects against P-glycoprotein (P-gp) overexpressing in multidrug-resistant cancer cells, has been a well-behaved carrier material for antineoplastic agents [54,57,58]. Nanoparticles utilizing TPGS individually to encapsulate CUR were regarded as an effective and safe delivery platform for oral administration, which were able to avoid degradation of the drug in the gastrointestinal tract and could be used for the treatment of colorectal cancer [57]. Despite its beneficial potential, the utilization of TPGS was still restricted due to high CMC and poor anti-dilution ability, making it difficult to maintain stability in blood circulation when administered intravenously. In view of these properties, F127 and P123 were added into the micellar system to prepare a CUR-loaded polymer-mixed micelle (CUR@NPT100), which held promise for the treatment of cervical cancer [54]. On the one hand, from the properties of the polymer carrier materials, the CMC values of the mixed copolymer and simplex TPGS were approximately 0.02 and 0.2 mg/mL, respectively, indicating that the copolymer micelles were expected to have great stability after dilution in the blood stream. On the other hand, from the perspective of efficacy, compared with non-cancerous cells NIH3T3, the mixed micelle significantly promoted the selective uptake of CUR by cervical cancer cells HeLa. Therefore, at the same drug concentration (2 μg/mL) for 48 h, CUR@NPT100 did not show evident cytotoxicity to NIH3T3 cells (the cell viability was approximately 85%) and showed a strong inhibitory effect on HeLa cells (the cell viability was about 55%). Moreover, the addition of TPGS to polymeric micelles can also significantly improve the encapsulation efficiency and bioavailability of drugs. When Soluplus/TPGS (3:2) were used as the carrier materials instead of Soluplus alone, the encapsulation efficiency of diosgenin increased from 66.7% to 92.6%, and the drug-loading rate increased from 3.3% to 4.6% [47]. When appropriate amounts of TPGS were added into HA-SS-PLA/PTX micelles, the t_1/2_, MRT, AUC_0–∞_, and peak concentration (C_max_) were raised by a factor of 1.33, 1.53, 2.05, and 1.33, respectively, indicating that mixed micelles could increase the retention time of simplex polymer micelles in vivo and improve the bioavailability of drugs [59]. Polymer micelles have attractive flexibility in design and can be composed of copolymers with multifarious physical and chemical properties, which is perfectly suitable for drugs with different degrees of hydrophobicity. Currently applied in establishing drug delivery system, polymer micelles have more practical meanings and can be considered as an ideal drug administration strategy against cancer.

#### 2.1.2. Liposome

Liposomes, which can encapsulate or incorporate drugs into lipid bilayers, have many superiorities, such as sustained release, low toxicity, high stability, and strong permeability [60,61,62]. Once in the bloodstream, conventional liposomes would be coated with a series of plasma proteins, such as immunoglobulins and complements, giving rise to enhanced affinity with mononuclear macrophages, which would make it easy to be cleared in systemic circulation and prevent it from exerting long-lasting effects [63,64]. Therefore, researchers have been searching for more suitable carrier materials with the aim to obtain long-circulating function.

On the one hand, liposomes can achieve long-term circulation function through biological modifications. Erythrocytes, a type of circulating cell, has great biocompatibility, biodegradability, and long circulation properties. An increasing number of studies have confirmed that nanocarriers coated with erythrocyte membranes had preponderances in terms of long circulation and biocompatibility [65,66,67]. Zhong et al. [68] designed a novel biomimetic liposome coated with erythrocyte membranes and a co-loading of triptolide and celastrol (C + T/RBCm@Lip), which could effectively evade recognition and clearance by macrophages. Erythrocyte membrane coating could not only avoid the rapid clearance of an immune system and prolong the blood circulation of liposomes but also increase the uptake of liposomes by tumor cells and enhance the inhibitory effects of the drug on the growth of HepG2 cells (compared with free drugs, the inhibition activity of two drugs encapsulated in C + T/RBCm@Lip both decreased by a factor of 1.18). In addition to erythrocyte membranes, the coating of bovine serum albumin (BSA) also can endow ordinary liposomes with long circulation function. Wei et al. [69] prepared CUR liposomes with a BSA coating (BSA-CUR-Lips). It was found that the phagocytosis of BSA-CUR-Lips by the mouse macrophage Raw 264 was significantly reduced (*p* < 0.05), indicating that the liposomes could exert long-circulating effects. In addition, BSA-coated nano-carriers also have potential values for applications in bioimaging [70].

On the other hand, liposomes can be modified with structure-specific chemicals to obtain long-circulating effects. Utilizing 2-distearoyl-sn-glycero-3-phosphoethanolamine-N-methoxy-PEG2000 (DSPE-PEG2000) to modify liposomes has become a research hotspot over the years. DSPE-PEG2000, causing powerful steric hindrance and hydrophilicity in liposome systems, can prevent liposomes from binding to plasma opsonin or being ingested by monocytes and macrophages [63,71,72]. Long-circulating liposomes are much more suitable for diseases that require long-term and frequent administration and therefore have promising applications in cancer chemotherapy.

Despite the long-circulating effect and better pharmacological efficacy of such liposome, high doses of cholesterol, which play the key role of membrane stabilization, have severely hindered the clinical application of liposome in cancer patients who also suffer from concomitant hyperlipidemia and cardia-cerebrovascular diseases [73,74,75,76]. Moreover, the use of cholesterol also refers to religion and vegetarianism [76]. Numerous methods have been explored to address these issues. β-sitosterol succinic anhydride ester, a potential alternative drug delivery carrier for cholesterol, was linked to PEG2000 and applied to the preparation of a gambogic acid liposome [63]. While possessing more outstanding long-term circulation effects than ordinary long-circulating liposomes (compared with the ordinary long-circulating liposomes, the t_1/2_ and AUC of the novel liposomes were increased by 12.5% and 47.1%, respectively), this novel long-circulating liposome could also remedy the deficiency of cholesterol.

Ginsenosides are a class of compounds with both hydrophilicity and hydrophobicity, in which the hydrophobic domain is equipped with the same steroid structure as cholesterol, and the hydrophilic domain is constituted by two glucose groups [77,78]. First of all, from the perspective of carrier structure, the ginsenosides Rg3 and Rb2 can not only substitute cholesterol for exerting a membrane stabilizing effect but also act as long-circulating stealther instead of DSPE-PEG2000 [76,79]. Additionally, in the sight of therapeutic effects, when combined with chemotherapy drugs, the ginsenosides Rg3 and Rb2 can exert synergistic anti-cancer effects [79,80,81,82].

It can be seen that the design of nanocarriers is constantly being updated, and numerous attempts have been made to design for safer and more effective ways to prepare nanocarriers with excellent characteristics such as high stability, long circulation, more powerful efficacy, and so on. Natural medicines have found their way here, which can be used not only as an alternative or synergist for traditional chemotherapy agent but also a pharmaceutical excipient in the production of anti-cancer preparation. The exploration of nano-preparation of natural medicines is meaningful and worthy of further study.

#### 2.1.3. Nanoemulsion

Nanoemulsions are thermodynamically stable colloidal solutions formed by droplets of the internal phase, with a particle size of 50 to 100 nm dispersed in in the external phase [83]. There are two major administration routes in the use of nanoemulsion: transdermal administration and oral administration [84,85]. As an ideal drug delivery carrier, nanoemulsions can increase the solubility, bioavailability, and anti-tumor activities of drugs [86]. At present, there are three main ways for nanoemulsions to carry natural medicines. Firstly, monomeric compounds with anti-tumor activities isolated from natural medicines were directly encapsulated using nanoemulsion technology [87,88]. In one study, luteolin was encapsulated into a nanoemulsion for the treatment of breast cancer. Due to its ability to improve the permeability of the skin stratum corneum, such nanoemulsions could directly deliver drugs to the tumor site through transdermal administration [87]. Secondly, aqueous solutions of polysaccharides in natural medicines are usually used as aqueous phases to prepare nanoemulsions. Li et al. [85] prepared a water-in-oil (W/O) nanoemulsion of shiitake mushroom polysaccharide (SMP), which was able to maintain relatively stable droplet size for 3 months (storage conditions were 4 °C or 37 °C), and the intestinal absorption and anti-tumor activity of SMP were clearly improved (anti-tumor activity for 18-fold, compared to non-treated SMP). Thirdly, using essential oils of natural medicines as the oil phase to prepare a nanoemulsion can not only improve the solubility and bio-accessibility of an essential oil, fully exert its anti-tumor effects, and expand its application range, but also avoid the potential toxicity caused by conventional oil phases [89,90,91]. Alam et al. [91] prepared a nanoemulsion system with high stability using cinnamon essential oil both as antineoplastic agent and as oil phase. Compared with cinnamon essential oil, the cytotoxic effect of nanoemulsion against A549 cells was significantly improved, with the IC50 decreased by a factor of 2.77. The physical properties and anti-tumor effects of some natural medicines nanoemulsions are shown in Table 2.

In addition, compared with other forms of nanoemulsions, there are few studies on the preparation of nanoemulsions from aqueous polysaccharide solutions. It has been reported that polysaccharides in other natural medicines also possessed anti-tumor effects, such as Angelica sinensis polysaccharides [92], Dendrobium wardianum polysaccharides [93], and Poria cocos polysaccharides [94]. However, few researchers have made them into nanoemulsions. Therefore, it may be a novel and promising research direction to prepare nanoemulsions using polysaccharides in natural medicines, which is expected to expand the application scope of polysaccharides and improve their anti-tumor activities.

**Table 2:** The physical properties and antitumor effects of some natural medicine nanoemulsions.

Natural Products	Oily Phase	Surfactant	EE (%)	DL (%)	Zeta Potential (mV)	Size (nm)	Stability	Tumor Cell	IC50		Ref.
									Free Drug	Nanoemulsion	
Paclitaxel	Coix seed oil	Kolliphor^®^ HS 15	98.8	0.978	−4.40 ± 1.13	30.28 ± 0.36	30 days	HeLa	3.101 ± 0.0375 µg/mL	1.378 ± 0.0230 µg/mL	[90]
Quercetin (Q) and curcumin (C)	Soy lecithin	Polysorbate 80	Q: 88.83 C: 85.37	Q: 0.71 C: 0.83	+26	25.9 ± 1.59	6 months	MCF-7	-	21.23 ± 2.16 µM	[95]
Mushroom polysaccharide	Isopropyl tetradecate	Tween 80 and Span 85	97.81	-	-	144.5	3 months	K562	4.22 mg/mL	0.235 mg/mL	[85]
Pomegranate polysaccharides	Glycerylmonooleate	Cremophor RH 40	92.82	-	−30.6	9.5	-	HCT-116	287.5 µg/ml	125.75 µg/mL	[96]
Quercetin	Olive oil	Span 60 and Tween 80	-	-	−53.7 ± 0.52	21.7 ± 1.6	-	HepG2	33.8 mM	23.4 mM	[88]
Costunolide	Pumpkin oil	Replace with α-CD	-	-	-	199.56	-	A549	13.4 ± 1.5 µM	6.1 ± 0.8 µM	[97]
Zingiber ottensii essential oil		Tween 80	-	-	−4.44 ± 0.92	13.8 ± 0.2	-	A549	43.37 ± 6.69 ng/mL	18.45 ± 3.33 ng/mL	[89]
								MCF-7	9.77 ± 1.61 ng/mL	1.08 ± 2.58 ng/mL	
								HeLa	23.25 ± 7.73 ng/mL	5.81 ± 2.38 ng/mL	
								K562	60.49 ± 9.41 ng/mL	32.48 ± 1.21 ng/mL	
Heracleum persicum essential oil		Polysorbate 20 and 80, Tween 80	-	-	−47.9	153	-	MDA-MB-231	-	2.32 µg/mL	[98]
Citronella essential oil		Tween 20	95.5 ± 4.775	-	−12.6	130 ± 5	30 days	A549	41.20 µg/mL	37.71 µg/mL	[99]
Cinnamomum cassia essential oil		Polysorbate 80	63.65 ± 3.182	-	−5.6	221.8	30 days	A549	50.21 µg/mL	18.05 µg/mL	[91]

Notes: EE represents encapsulation efficiency. DL represents drug-loading rate. IC50 represents half maximal inhibitory concentration of a drug.

### 2.2. Active Ingredients in Natural Product-Mediated Drug Targeting

**Figure 4:**
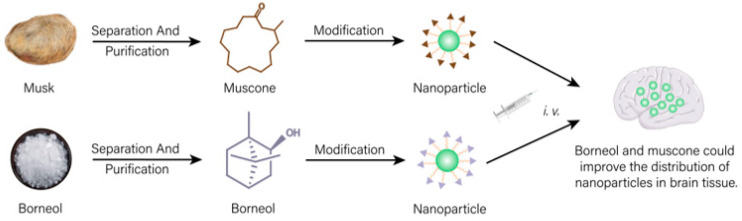
Drug delivery system modified with effective ingredients of natural medicines could increase distribution of other drugs in targeted tissue.

In natural medicines, there is a subset of drugs that are similar to targeting formulations in modern medicine and normally used to achieve “site-directed” effects [100]. As shown in Figure 4, a drug transportation system modified with active ingredients of natural medicines could change the action site of other drugs and increase the distribution of drugs in targeted tissues, which have important application values in nano-formulations. 

Borneol and musk, belonging to aromatic resuscitation herbs in natural medicines, can be used to modify nano-preparations acting on the brain, which can increase drug distribution in the brain and improve the brain targeting of the formulation [101,102,103]. Borneol (Bor) and transmembrane peptide Pep-1 co-modified micelles loaded with carmostine exhibited good therapeutic effects on brain glioma. Bor could effectively improve the blood–brain barrier (BBB) permeability for drugs. Cell experiments showed that the modification of Bor could not only remarkably enhance the cytotoxicity of drugs on human glioma BT325 cells (*p* < 0.01) but also evidently increase the uptake of micelles by brain microvascular endothelial cell lines HBMEC (fluorescence intensity increased 1.67 times). In vivo experiments showed that the fluorescence of untargeted micelles labeled with fluorescent probe DiD disappeared rapidly at 6 h post-injection. A single dose of Bor-modified micelles significantly increased the signal distribution in brain tissue (*p* < 0.01), suggesting that Bor modification enhanced its ability to penetrate the BBB. Additionally, the signal was still observed 24 h after treatment, indicating prolonged retention times in in brain tissue [101]. Other studies showed that similar to Bor, due to the brain targeting property of muscone, doxorubicin-loaded liposomes modified with muscone could cross the BBB. Compared with unmodified liposomes, muscone-modified liposomes showed concentrated accumulation in the glioma region of the brain and less distribution outside the glioma region. Thus, muskone modification could increase the distribution of drugs in brain tissue, then improving the curative effect for brain glioma of antineoplastic drug [103].

### 2.3. Ligand-Mediated Drug Targeting

#### 2.3.1. APRPG Peptide Modified Nanocarrier

Vascular endothelial growth factor (VEGF) is highly expressed on tumor vascular endothelial cells but rarely on normal endothelial cells [104,105], making it a desirable target point for anti-tumor drug delivery. Ala-Pro-Arg-Pro-Gly (APRPG), the small molecule peptide sequence, is able to specifically bind to VEGF receptors (VEGFR). The modification of APRPG on the nano-formulations can considerably increase the effect of the first-level drug targeting by means of active targeting. It can actively deliver the drug to the tumor tissue and improve the effectiveness of chemotherapy [106,107,108]. After intravenous administration, APRPG-modified nanoparticles loaded with PTX and norethindrone were concentrated at the cancer site of mice and effectively inhibited the growth of ectopic solid tumors in tumor-bearing mice (the inhibition rate of APRPPG-modified nanoparticles group and non-targeted nanoparticles group were 78.67% and 62.98%, respectively) (*p* < 0.01), suggesting that APRPG could deliver chemotherapeutic drugs more effectively to tumor tissues through active targeting [108].

It is important to note that VEGF is not only a target for drug delivery but also the site of action of natural anti-tumor monomer components. For example, triptolide restrained breast cancer cell angiogenesis through inhibiting the ERK1/2-HIF1-α-VEGFA axis [109]. Honokiol inhibited the NF-κB pathway, which, in turn, led to the down-regulation of VEGF expression and reduced the viability and angiogenesis of human lung cancer cell lines [110]. Cantharidin inhibited tumor angiogenesis by suppressing VEGF-induced signaling pathways [111]. The development of angiogenesis inhibitors targeting VEGF/VEGFR has become a vital field in anti-tumor research. Therefore, when designing nano-targeted agents, we can fully consider the site of action of drugs according to the therapeutic purpose and select the most appropriate drug for delivery.

#### 2.3.2. NGR Peptide-Modified Nanocarrier

NGR peptide (NGR), a peptide containing an asparagine-glycine-arginine (Asn-Gly-Arg) motif, is capable of specifically recognizing aminopeptidase N (APN/CD13), which is highly expressed on tumor vascular cells [112,113,114]. NGR is considered as a potential targeting ligand that can target tumor blood vessels [112]. Therefore, similar to APRPG, NGR ligands can be used for surface modifications of nano-formulations to enhance the targeting ability of drug delivery systems to tumor tissues [115,116].

This targeted ligand can not only increase the effect of first-level drug targeting depending on the means of active targeting, so that chemotherapeutic drugs or nano-agents with anti-tumor effects can accumulate more effectively and selectively in cancer tissues and thus be more fully taken up by tumor cells. It can also make drugs that have the ability to regulate the tumor environment accurately locate in tumor tissue and act as an anti-tumor agent indirectly by improving the tumor environment. A dual-targeted micelle-liposome bilayer delivery platform triggered by matrix metalloproteinases was designed by Duan et al. [115] for simultaneous loading of the anti-fibrotic drug quercetin (Que) and the herbal chemotherapeutic drug PTX. Owing to the first-level drug targeting function of NGR, the liposomes carrying Que on the outer layer of the formulation specifically accumulate at the tumor tissue to exert the anti-fibrotic effect of Que and ameliorate the tumor microenvironment. Modification of NGR evidently facilitated intracellular accumulation of liposomes in human umbilical vein cell lines (HUVEC), with an uptake 1.3 times higher than that of unmodified liposomes. This suggested that the NGR-modified formulation could function to target tumor tissue through CD13 receptor-mediated endocytosis. The results of in vivo experiments exhibited that non-targeted agents were rapidly eliminated after injection, whereas NGR modification not only significantly increased the accumulation of the agents at the cancer site (*p* < 0.05) but also distinctly prolonged the residence time of nanoparticles at the tumor site (fluorescent signals were still detectable after intravenous injection for 24 h), suggesting that NGR played a crucial role in mediating the accumulation of nano-agents in tumor tissue.

It is worth noting that the above two targets and pathways initially are not used for the first-level drug targeting but for specifically delivering anti-angiogenic drugs to tumor blood vessels for the purpose of tumor therapy by inhibiting the generation of new blood vessels [112,117]. But now, NGR and APRPG have found their new way to achieve a tumor tissue-specific targeting function as an active targeting ligand. This also provides inspiration and ideas for our future research: due to the difficulty in discovering new targets and pathways, when designing new targeting carriers, the flexibility of a nanocarrier can be fully utilized. Starting from known targets and pathways, new carrier forms can be explored to obtain more novel and powerful targeting strategies and expand the application range of natural medicines’ nano-formulations.

## 3. Second-Level (Cell-Specific) Drug Targeting

Although the drug delivery systems mediated by first-level targeting can deliver drugs to the tumor site, they still suffer from poor tumor cell specificity. There are evident discrepancies in the receptors’ expression, metabolism, or other biological properties between cancer and normal cells. It has enormous potential for anti-tumor treatment to switch these differences to advantages to ensure that drugs are more easily and accurately transported to tumor cells to exert their anti-tumor effects [118,119].

### 3.1. Folate Modified Nanocarrier

The folate receptor (FR) is a type of glycoprotein anchored on the cell membrane by glycosylphosphatidylinositol (GPI) and has a high affinity with folate (FA) (K_D_ = 10^−10^ M) [120]. FRs are overexpressed on various types of tumor cells, and their activities are also significantly higher than those of normal cells. FRs are divided into three subtypes, α-FR, β-FR, and γ-FR. α-FR is highly expressed in some tumor cells such as ovarian cancer, cervical cancer, breast cancer, lung cancer, kidney cancer, colorectal cancer, and brain tumors; β-FR is overexpressed on the surface of placenta, mature neutrophils, and myeloid leukemia cells; and γ-FR is mainly overexpressed in malignant leukemia cells [121,122]. By utilizing the difference in FA receptor expression between tumor and normal cells, FA-modified drugs can easily enter cancer cells through endocytosis mediated by FR. Song et al. [123] prepared FA modified nanoemulsions (FNEs) with high drug contents and excellent tumor cell targeting abilities, which can increase the accumulation of PTX in tumor cells (compared to unmodified nanoemulsions, the intracellular fluorescence intensity increased by approximately 71.4%), effectively inhibiting tumor growth (the tumor volumes were reduced by more than 25%), prolonging survival times (50% of mices receiving FNEs survived for 60 days during the experiment), and having low toxicity to normal tissues (no significant weight loss or histological abnormalities of major organs were discovered).

The targeting efficiency of nano-formulations is influenced by external and internal factors. Therefore, researchers continuously develop new methods to avoid or optimize these factors. In this regard, many studies have focused on FA.

On the one hand, the content of targeted ligands in a carrier material has a significant impact on the targeting effect. Either too little or too many ligands can be detrimental to the tumor-targeting capability of the nano-formulation. Grigletto et al. [122] found that 3% FA conjugates (molar content) were able to block the cell cycle of 85% of HT-29 cells in G2/M phase, whereas 1% FA conjugates and PTX-PEG (none of FA conjugates) were only able to block approximately 20% of cells. Another study documented that FA-F127-PCL micelles conjugated with 10% FA exhibited stronger cytotoxicity and higher cell uptake rate on ovarian cancer cell line OVCAR-3 compared to 50% and 91% FA micelles [124]. This might be because excessive FA groups on the surface of nanoparticles tended to produce the steric hindrance effect, which, in turn, limited their targeting ability. Therefore, there should be an optimal range of the content of FA groups on the surfaces of nanoparticles to achieve preferable targeting towards cancer cells. This also provides insights for the usage of other targeted ligands: the optimal range of different types of ligands for different types of tumor cells still needs further research to achieve the maximum targeting effect.

On the other hand, the efficiency of active targeting is also influenced by the external environment. When nanoparticles enter the physiological environment, biological macromolecules in blood stream such as proteins will spontaneously adsorb on the surface of nanoparticles. This phenomenon is known as the protein coronas (PCs) effect. The formation of PCs would weaken the targeting capability of the agent [125,126]. Studies have revealed that red blood cell membrane could be used as coatings, which was able to effectively refrain from the impact of PCs on the original biological properties of nanoparticles [126]. When nano-preparations conjugated with FA were exposed to biological fluids, FA molecules on the surface were covered with PCs, leading to a sharp decrease on the ability of the nanoparticles to bind to the FR (*p* < 0.001), and thus their targeting ability to human breast cancer cells MCF-7 was also reduced. On the contrary, the targeting efficiency of nanoparticles was remarkably improved after coated with red blood cell membranes, which might be attributed to the lack of PCs. Therefore, another efficient approach to improve a ligand’s targeting ability is selecting appropriate materials to modify formulations to avoid PC effects, which can be applied to the design and preparation of active targeting drug delivery systems for natural medicines to better deliver drug to tumor cells and exert anti-tumor effects.

### 3.2. Transferrin Modified Nanocarrier

Although the transferrin receptor (Tf-R) is widely distributed in the body, its expression on the surface of tumor cells is significantly enhanced. Additionally, the affinity of transferrin (Tf) to Tf-R on the surface of tumor cells is dozens of times higher than that of normal cells [127]. In order to improve the cell-specific targeting ability of nano-formulation, Tf can be modified onto nanocarriers as a functional group. Liu et al. [128] designed a liposome-encapsulated syringic acid (CA) (LP-CA) and modified it with Tf (Tf-LP-CA) for the purpose of promoting the uptake of the nanoparticles by tumor cells and improving the anti-tumor effect of chemotherapeutic drugs. In HepG2 cells, compared with the free drug and unmodified liposome, the IC50 value of Tf-LP-CA decreased by a factor of 2.72 and 1.32, respectively. Likewise, the IC50 value of Tf-LP-CA decreased by a factor of 2.52 and 1.85, respectively, in SMMC-7721 cells. It was also exhibited that the uptake of Tf-LP-CA on liver cancer cells HepG2 (*p* < 0.05) and SMMC-7721 (*p* < 0.01) was significantly higher than that of LP-CA, whereas there was no significant difference between the uptake of LP-CA and Tf-LP-CA by normal liver cells L-02, suggesting that Tf-LP-CA targeting is primarily through the Tf-R. Animal experiments showed that Tf-LP-CA was mainly distributed in the liver of mice and could inhibit the growth of ectopic solid tumors in mice bearing liver cancer cells (*p* < 0.05). Li et al. [129] constructed Tf modified nanoparticles (Tf-PIP-NPs) for the delivery of piperine. Tf-PIP-NPs exhibited more potent cytotoxicity and apoptotic effects against a variety of tumor cells, including HepG2, MDA-MB-231, and 4T1. After the 72 h administration, compared with unmodified liposomes, IC50 value of Tf-PIP-NPs decreased by a factor of 10.2, 2.4, and 1.2 times in HepG2, MDA-MB-231, and 4T1 cells, respectively. Similarly, in the cell apoptosis experiment, the ability of the preparation to induce cell apoptosis was 3.04, 2.03, and 1.34 times, higher than that of the free drug, respectively, in the three types of cells mentioned above. In the 4T1 tumor-bearing mouse model, after 1 h of administration, nanoparticles were rapidly taken in by tumor cells through the Tf-R mediated endocytosis pathway to exert anti-tumor effects.

### 3.3. Hyaluronic Acid Modified Nanocarrier

Cluster of differentiation-44 (CD44) receptors are overexpressed in many types of tumor cells, such as breast cancer, pancreatic cancer, colorectal cancer, ovarian cancer, brain cancer, lung cancer, etc. [130,131,132]. Hyaluronic acid (HA) is a negatively charged natural polysaccharide with high affinity for CD44 [133]. Therefore, modifying HA onto the surface of drug carriers can specifically target cancer cells with high CD44 receptors expression by utilizing CD44 receptor-mediated endocytosis [134,135,136,137]. Ji et al. [138] designed HA-modified nanocrystals for the encapsulation of CUR (HA@CUR-NCs) to treat breast cancer. Compared with free drugs and CUR-NC, HA@CUR-NCs not only promoted cell apoptosis (*p* < 0.01) and enhanced intracellular uptake (*p* < 0.001) in MDA-MB-231 cell lines but also extended the t_1/2_, MRT, and AUC_0–t_ of CUR and showed superior anti-cancer effects in the 4T1 in situ breast cancer model of mice. The t_1/2_, MRT, and AUC_0–t_ of the HA@Cur-NCs were almost 4.8-, 5.6-, and 6.3-fold higher than that of Cur, respectively. After 10 days of administration, the tumor volume of the tumor-bearing mice significantly decreased (*p* < 0.01), and the tumor weight decreased by nearly half.

### 3.4. Galactose Modified Nanocarrier

There are highly expressed asialoglycoprotein receptors (ASGP-R) on the membrane of liver cancer cells, which can specifically recognize and bind galactose (Gal) [46,139]. Therefore, utilizing Gal to modify drug carriers or drug molecules can target drugs to liver cancer cells, increase the liver targeted distribution efficiency of drugs, and improve the chemotherapy effects. In order to achieve accurate and effective liver-targeted delivery, galangin was encapsulated into a Gal-F68 polymer micelle that could increase the content of galangin in the liver by 7.18 fold compared to using galangin alone [46].

The hepatic targeting efficiency of nano-preparation improves with the increase in Gal concentration. Sun et al. [43] constructed Gal-modified polymeric mixed micelles for oral delivery of CUR (CUR@GPP). The fluorescence images of the main organs after oral administration for 24 h showed that, with the augment in Gal content, the fluorescence intensity of GPP micelles in the liver was significantly enhanced, whereas there was almost no signal in other organs, including the heart, spleen, lungs, and kidneys. Moreover, galactose lectin, the other transmembrane receptor of Gal, is abundantly expressed in the gastrointestinal tract. Gal modification also could increase the absorption of micelles in the jejunum and ileum. The relative bioavailability of CUR@GPP relative to micelles without targeting ligands was 258.8%. This demonstrated that Gal-modified nano-preparations could broaden the route of administration and improve patient compliance, as well as provide new research ideas and methods for the treatment of hepatocellular carcinoma.

### 3.5. Glycyrrhetinic Acid Modified Nanocarrier

There are abundant specific binding sites of glycyrrhetinic acid (GA) on the surface of hepatocellular carcinoma cells. By modifying the nano-carrier with GA, the liver targeting ability of the formulation can be greatly improved. Moreover, GA itself has certain anti-cancer and anti-inflammatory effects. Therefore, using GA as a ligand is a promising research direction for the development of targeted therapeutic drugs for hepatocellular carcinoma [140,141]. Song et al. [13] connected GA with F68 and mixed it with F68-acetal-PCL copolymer to prepare CUR polymer-mixed micelles (MIX/CUR). MIX/CUR could enhance the inhibitory effect of CUR on the growth of in hepatocellular carcinoma cells. In total, 48 h after administration, the IC50 value of MIX/CUR in HepG2, SMMC 7721, and Hepa1-6 cells decreased by a factor of 2.4, 2.3, and 3.0, respectively, compared with the free drug. In cellular uptake assays, intracellular fluorescence intensity of MIX/CUR increased by more than five times compared with non-targeted micelles, indicating that GA modification could enhance the uptake of the mixed micelles by hepatocellular carcinoma cells through GA receptor-mediated endocytosis. Zhu et al. [142] synthesized an amphiphilic derivative of GA, 3-succinyl-30-stearylglycyrrhetinic acid (18-GA-Suc), and used it as one of encapsulants to prepared cantharidin (CTD)-loaded liposomes (18-GA-Suc-CTD-Lip). Studies on the biological distribution of drugs showed that 18-GA-Suc-CTD-Lip was mainly distributed in the liver, and the concentration of it in the liver was significantly higher than that of CTD-Lip, within 90 min of administration. Especially, 18-GA-Suc-CTD-Lip reached its maximum concentration (1.72 ± 0.14 µg/g) at 30 min, which was more than twice that of CTD-Lip (0.75 ± 0.08 µg/g).

### 3.6. RGD Peptide Modified Nanocarrier

RGD peptide (RGD), a class of short peptides containing Arginine-Glycine-Aspartic acid (Arg-Gly-Asp) sequence, can specifically bind to integrin receptor αvβ3, which is overexpressed on the surface of various tumor cells, including glioma cells, ovarian cancer cells, gastric cancer cells, breast cancer cells, melanoma cells, etc. [143,144,145,146,147]. This advantage endows RGD with great talent as an active targeting ligand for accurate delivery of therapeutic agents. Long et al. [146] developed a novel delivery system of RGD-conjugated resveratrol human serum albumin (HAS) nanoparticles (RGD-RVT-HSA NPs) for ovarian cancer therapy. In such a nano-drug delivery system, RGD was modified on the surfaces of nanoparticles to penetrate cancer tissues and target cancer cells. HAS was used as the drug carrier. Resveratrol was encapsulated in nanoparticles as an anti-cancer drug. The results of cell experiments showed that uptake of RGD-RVT-HSA NPs in ovarian cancer cell lines SKOV3 was significantly higher than that of unmodified nanoparticles (approximately 3.6-fold higher), which might be related to the high expression levels of integrin receptors on the surface of SKOV3 cell. The results of in vivo studies displayed that RGD-RVT-HSA NPs could significantly accumulate in tumor tissue and could evidently reduce the size of solid tumor in mice. At the end of the study (27-day), compared with free drugs and unmodified nanoparticles, the tumor volumes of RGD-RVT-HSA NPs groups were reduced by 47% and 34%, respectively.

### 3.7. Glucosyl Group/Glucose Derivative Modified Nanocarrier

Glucose transporter (GLUT) can mediate glucose transmembrane transport. During cancer, tumor cells require a large amount of glucose to provide energy for rapid proliferation. Therefore, GLUT is abundantly expressed on the surface of tumor cells [148,149]. Recently, the design and development of tumor cells-targeted nano-preparation in virtue of GLUT-mediated endocytosis has become a hot research topic [149,150,151]. As modifying glucose with alkyl groups can enhance the affinity of glucose to GLUT, researchers used n-octyl-β-D-glucopyranosideis as a targeting ligand to modify liposomes loaded with dihydroartemisinin [151]. Compared with ordinary liposomes, it had a stronger targeting effect on HepG2 cells (*p* < 0.01).

In addition to the advantages mentioned above, ginsenosides can also act as active targeting ligand [76]. The glucose groups in ginsenoside have a strong affinity for GLUT and can deliver the agent to tumor cells via GLUT-mediated endocytosis [76,78]. Ginsenoside Rh2 liposome (Rh2-lipo), a novel type of liposome, was prepared by using paclitaxel as a model drug [76]. In the cell uptake experiment, Rh2-lipo exhibited higher fluorescence intensity than conventional liposomes (the fluorescence intensity increased by nearly 2.3-fold). In animal experiments, the 4T1 in situ tumor rat was used to evaluate the targeting effect of Rh2-lipo. After administering DiD-labeled liposomes, compared with the conventional liposome group, the Rh2-lipo group showed stronger fluorescence signals in the tumor area (the fluorescence intensity increased by nearly 4-fold), indicating that Rh2-lipo could evidently enhance the effective accumulation of liposomes at the tumor site.

### 3.8. Other Targeted Ligands Modified Nanocarriers

In addition to the targeting ligands mentioned above, chondroitin sulfate, lysine, and others also possess second-level targeting functions. Some second targeting ligands used in natural medicines nano-formulations are shown in Table 3. The ligand types and functions of some nano-formulations with second targeting function are shown in Table 4.

## 4. Third-Level (Organelle-Specific) Drug Targeting

### 4.1. Targeting to Mitochondria

Mitochondria play an indispensable role in energy metabolism, redox signalling, and programmed cell death [177]. The modification of lipophilic cations, including triphenylphosphine, berberine, etc., can enable nanocarriers to achieve mitochondrial targeting [178,179]. Taking advantage of the difference in mitochondrial membrane potential between tumor cells (−220 mV) and normal cells (−140 mV) [180], this nanoparticles is able to selectively deliver chemotherapy drugs to mitochondria across physiological barriers and then induce cell apoptosis [181].

Mitochondria are a key target for tumor chemotherapy. It has been found that many active ingredients of traditional Chinese medicine can exert anti-tumor effects through the mitochondrial pathway. For example, RES can affect the mitochondrial autophagy in non-small-cell lung cancer cells through the PINK1/Parkin pathway [182]. Triptolide can inhibit the growth of hepatocellular carcinoma through the mitochondrial apoptosis pathway [183]. Therefore, the design of drug delivery systems targeting mitochondria is crucial for the treatment of cancer.

#### 4.1.1. Triphenylphosphine Modified Nanocarrier

Triphenylphosphine (TPP), a non-localized cationic group composed of three benzene rings, is commonly used to mediate chemotherapeutic drugs or nano-preparations to overcome the obstruction of biomembranes and, subsequently, target mitochondria [20,184,185].

However, nanocarriers with TPP modification, which have positive charges on their surfaces, are easily cleared in the bloodstream. In light of this issue, HA and TPP co-modified nano-formulations for mitochondrial targeting have been subject to increasing interests during recent years [186,187,188]. On the one hand, HA can shield the positive charge of TPP to prolong circulation time of nano-carrier. On the other hand, HA-conjugated nanoparticles can target tumor cells via CD44 receptor-mediated endocytosis. In turn, HA is degraded under the action of hyaluronidase, and TPP at the inner layer is exposed, so as to stepwisely deliver drugs to mitochondria and, subsequently, improve the effectiveness and safety of the nanotherapeutic.

Recently, a polymeric nano-formulation with TPP-F127-HA as an encapsulant was developed (TPH/PTX) to conserve the anti-tumor activity of PTX before reaching the mitochondria [186]. After increasing the uptake of micelles by A549 cells, mitochondrial colocalization occurred, owing to the positive charge of TPP. TPH/PTX successively completed escape and mitochondrial targeting within 24 h. Then, it played an anti-tumor role by reducing the mitochondrial membrane potential, activating the apoptotic caspases 9/3, increasing the expression of pro-apoptotic protein Bcl-2, and reducing the expression of the anti-apoptotic protein Bax. In a nude mice xenograft model of the A549 lung cancer cells, significant tumor targeting and powerful anti-tumor efficacy of TPH/PTX micelles could also be manifested (with a tumor inhibition rate of 81.7% on day 29).

In addition, mitochondrial-targeted nanoparticles also have great potential for applications in tumor imaging and chemical photothermal therapy. Kim et al. [187] reported a stimulus responsive and dual targeting nanoparticles (FNPs-pDA@HA-TPP-CD-PTX). In this drug delivery system, HA and TPP were targeted to the tumor cell and mitochondria, respectively. Boric acid (BA) was used to connect the outer shell and inner core, whereas possessing pH and photothermal responsiveness. FNPs-pDA was used for photothermal therapy and biological imaging. The results of confocal laser scanning microscopy (CLSM) showed that after administration, the mitochondrial sites of MDA-MB-231 cells exhibited strong fluorescence signals, indicating that nanocomposites have potent mitochondrial targeting activity. In vivo studies showed that FNPs-pDA@HA-TPP-CD-PTX was able to be highly enriched in tumor tissue and localized in mitochondria. Under infrared irradiation, it could generate high temperature and regulate the release of PTX, effectively inhibiting the growth of tumor cells and protecting surrounding healthy tissues from drug side effects. This drug delivery system was expected to provide support for the development of future imaging-guided chemotherapeutic drug delivery.

#### 4.1.2. Berberine Modified Nanocarrier

Berberine (BBR), an active component of natural medicines, possesses delocalized positive charges and amphiphilic properties. As a mitochondrial targeting ligand, it has higher biological safety compared to the other ligands [179,189,190]. Recently, by linking BBR and PTX via a disulfide bond, a novel reduction-sensitive nanoparticle (PTX-SS-BBR NPs) was prepared [190]. PTX-SS-BBR NPs, which could target to mitochondria of A549 cells, displayed higher anti-tumor effectiveness in vitro than the free PTX. Especially after 48 h treatment, the inhibition activity of PTX-SS-BBR NPs (IC50 = 0.243 μM) increased by approximately 22% compared to PTX (IC50 = 0.31 μM). Additionally, modifying BBR with lipophilic alkyl groups could improve its permeability to cell membranes, thereby enhancing its mitochondrial targeting ability. Song et al. [179] constructed mitochondrial targeting liposomes (HA/PEG/BD NDs) using 9-*O*-octadecyl substituted a berberine derivative (BD) as targeting ligand. It was found that HA/PEG/BD NDs could induce apoptosis in tumor cells by dissipating mitochondria membrane potential and releasing cytochrome C (compared to free drugs, the proapoptotic ability of HA/PEG/BD NDs increased by 2.12 times). In the A549 cell transplantation tumor model, HA/PEG/BD NDs also showed significant mitochondrial targeting, which were accordant with its anti-tumor effect in vitro. Moreover, the HA/PEG/BD NDs group showed significant inhibition on tumor growth. On day 27 of treatment, the HA/PEG/BD NDs groups achieved the most powerful tumor inhibition (61.7%), which was 6.19- and 1.58-folds higher than that of free drug and PEG/BD NDs groups, respectively.

#### 4.1.3. OPDMA Modified Nanocarrier

Although promising, it is noteworthy that the TPP or BBR modified materials are usually positively charged, which can result in strong non-specific binding between nanoparticles and serum proteins and lead to the rapid blood clearance of nano-drugs. Therefore, when applied, they tend to be modified to shield positive charges and maintain their stability in the systemic circulation, which increases the complexity of the nanosystem [179,191,192]. To counter this issue, the development of novel carrier material is possible. Poly(2-(*N*-oxide*N*,*N*-dimethylamino)ethyl methacrylate) (OPDMA) is an electroneutral polymer possessing mitochondrial targeting ability [192]. In a study, OPDMA was conjugated with celastrol, a natural product with anticancer capability, to prepare nanoparticles. Such nanoparticles possessing OPDMA shell could be quickly internalized by tumor cells, specifically accumulating in mitochondria, evidently repressing mitochondrial membrane potential and causing much potent cell lethality and apoptosis-promoting effects on a variety of cancer cells, including 4 T1, B16F10, HepG-2, HeLa, and A549 cells.

### 4.2. Targeting to Nucleus

It is generally recognized that DNA is the crucial target of a large number of anti-tumor agents. DNA topoisomerase (Topo), an important enzyme that exists in the nucleus, has received extensive attention in the field of cancer therapy [193]. It can be divided into Topo Ⅰ and Ⅱ, which play an important role in DNA transcription, replication, and gene expression [194]. A large number of active compounds isolated from natural products are known to be capable of inhibiting DNA Topo. For example, camptothecin and HCPT showed potent anti-tumor activity by targeting intracellular Topo I [195,196]. Curcumin was able to induce Topo–DNA complexes in tumor cells with both Topo I and II [197]. Flavonoids, such as apigenin, kaempferol, and quercetin, could be transformed into quinones through oxidation reaction in vivo and then interact with the DNA Topo I complex to inhibit its activity [194,198]. Although many anti-tumor drugs acting on the nucleus have been developed, the efficacy of these drugs has been greatly reduced due to poor targeting and strong toxic side effects. The novel platform for delivering chemotherapy drugs to the nucleus of tumor cells have been the research focus and received much attention in recent years.

#### 4.2.1. Nuclear Localization Signal Peptide Modified Nanocarrier

Nuclear localization signal peptide (NLS), as a functional polypeptide, can interact with α and β input proteins and then localize in the nucleus through the nuclear pore complex by consuming GTP [199,200]. The application of NLS in the construction of a functional nano-drug delivery system with nuclear targeting capability has great development potential in the field of anti-tumor therapy. The most widely studied NLS functional sequence currently is Lys-Lys-Lys-Arg-Lys (KKKRK) [201,202]. Yan et al. [202] designed a novel nuclear targeting nanocarrier composed of Kala peptide (Kala), NLS, and stearic acid (SA) (NLS-Kala-SA, NKSN). Due to the nuclear-targeting function of NLS, NLS-Kala-SA mainly accumulated in the nucleus of A549 cells and was mainly distributed in the tumor site of A549 tumor-bearing mice. The therapeutic effect of nanoparticles on tumor-bearing mice was superior to that of the free drug PTX (the relative tumor volume was reduced by 2.2-fold). This nano-system not only had nuclear targeting property but also had a wide applicability with high encapsulation efficiency for CUR, PTX, and ginsenoside K. In addition, Kala in the formulation was a cationic membrane-penetrating peptide that could facilitate drug transmembrane transport and clear biofilm barriers for the nuclear targeting of the formulation.

#### 4.2.2. Macrocyclic Polyamine Modified Nanocarrier

1,4,7,10-tetraazacyclododecane (Cyclen) is a type of macrocyclic polyamines. It has a high degree of protonation in the tumor environment at low pH and has a high affinity for negatively charged nuclei. Using Cyclen to modify nanoparticles can increase the accumulation of nano-agents in the nucleus [203,204,205]. Guo et al. [204] linked the anticancer drug HCPT with Cyclen and developed a supramolecular hydrogel (HCPT-FFFK Cyclen). The results showed that after administration for 4 h, the intracellular concentration of HCPT at HCPT-FFFK-cyclen group was approximately 2-fold compared with other groups. It indicated that HCPT-FFFK-Cyclen significantly improved the uptake of the drug and increased nuclear accumulation of drug in A549 cells compared to the free HCPT due to Cyclen’s function of targeting the nucleus. In addition, Cyclen was able to reverse the ATP-dependent drug efflux by hydrolyzing ATP in the cells, increasing the accumulation of the drug in the cells and further improving the drug efficacy. In vivo experiments showed that HCPT-FFFK-Cyclen was able to reduce the tumor volume of tumor-bearing mice compared with free drug (*p* < 0.01) and had no significant toxicity to healthy mice and other organs of tumor-bearing mice.

### 4.3. Targeting to Lysosome

Lysosome, an organelle with a single-layer membrane structure, plays an important role in recycling cellular waste and transmitting cell signals. Its low pH environment (pH 4–5) can activate a variety of hydrolases. While providing biomolecules and energy for rapid tumor growth, lysosome can also participate in tumorigenesis and development by regulating tumor cell autophagy, apoptosis, and so on, which are potentially important targets for tumor therapy [206].

Most lysosomal targeting ligands have small molecule lipophilic amine structures (neutral red, dimethylamine, morpholine, etc.), which can utilize pH as a driving force to achieve lysosomal targeting [207]. Some effective ingredients of natural medicines, such as triptolide, CUR, and PTX, can act on lysosomes, causing changes in the permeability of the lysosomal membrane and then lysosomal enzymes penetrate into the cytoplasm and induce apoptosis of cancer cells [208,209,210]. However, the poor selectivity of these small molecule chemotherapy drugs restricts their application in cancer treatment. For the purpose of improving the lysosomal targeting and anti-tumor effect of CUR, Wang et al. [211] prepared Gal- and morpholine (Mor)-modified dual-targeting CUR liposomes (Gal-Mor-LPs). Due to the attraction of acidic lysosomes to basic Mor groups, Gal-Mor-LPs showcased a better lysosomal targeting effect than conventional liposomes (LPS) and Gal-modified liposomes (Gal-LPs). The ability of liposomes to target hepatocellular carcinoma cells in vitro and in vivo and the anti-tumor effect showed a trend in LPs < Gal-LPs < Gal-Mor-LPs, indicating that dual targeting nano-carriers could improve the tumor suppressive effect of CUR.

It is worth noting that despite the promising application prospects of lysosomal targeted therapy, the research on nano-preparation-targeting lysosomes of natural medicines active ingredients has not received widespread attention, like nuclear- or mitochondrial-targeted therapy, possibly due to the following reasons: (1) lack of research on the mechanism of natural medicines effective constituents targeting tumors through the lysosomal pathway; (2) the study of relevant targeting mechanisms is not particularly sufficient; and (3) some therapeutic drugs are intolerant to acidic lysosomal microenvironments. Therefore, it is necessary to conduct more research on the lysosomal-targeted therapy of effective ingredients in natural medicines.

## 5. Conclusions

In recent years, the incidence rate and mortality of cancer have increased annually, and the existing medicines and treatment modalities are sometimes unable to satisfy the therapeutic needs of cancer. A large number of natural products have potent anti-tumor activities and are under preclinical studies. For example, genistein had a powerful therapeutic effect in vivo on A431 and Colo205 tumor-bearing mice [212]. De Oliveira et al. [213] selected 18 studies (116 animals) for meta-analysis. The results showed the evidence that CUR-loaded polymeric nanocapsules inhibit tumor growth (*p*  <  0.00001) and decrease tumor weight (*p* = 0.0006) in rodents was established. Natural polyphenols, such as apigenin and kaempferol, had therapeutic effects on colorectal adenoma by regulating different signaling pathways and targets [214]. These natural products are all effective and promising candidates for the treatment of cancer and are expected to be introduced into medical practice in the future. The combination of natural medicines and nano-targeting technology would be a vital development direction of natural medicine preparations with broad application prospects. This article reviewed the application of nano-targeting technology in anti-tumor preparations of natural medicines from the perspective of different targeting levels. Researchers could choose the most appropriate level of drug targeting according to clinical needs or drug properties.

Nano-targeting nanotechnology has promoted the innovation of natural medicine’s anti-tumor preparations, and there is still great room for improvement in their research and development:

1. The optimal usage contents of different targeted ligands still need further research;
2. In a further study of a nano-targeted drug delivery system, more attention should be paid to the combination of drugs acting on different targets. Ligands targeting different sites could be combined to design double-layer or multifunctional formulations, achieving programmed drug release and accurate drug delivery. There are relatively few studies on this aspect. Some multiple levels targeting of nano-preparations of natural medicines are shown in Table 5;
3. At present, the research on targeted formulations of natural medicines is mostly in the laboratory research stage, with few clinical applications. Therefore, when designing formulations, actual production requirements and clinical needs should also be taken into account to promote the industrial and clinical transformation of research results and better serve clinical needs;
4. Some targeted formulations have relatively low drug-loading capacities. Increasing the drug-loading capacity and reducing the number of doses is of great significance in reducing a drug’s toxicity and side effects.

## Figures and Tables

**Table 3:** Some second-level targeting factors applied to natural medicines.

Receptors	Targeting Ligands	Ref.
Folate (FA) receptor	FA	[152]
Transferrin (Tf) receptor	Tf	[129]
	T7 peptide	[153]
Cluster of differentiation-44 (CD44) receptor	Hyaluronic acid (HA)	[134]
	Chondroitin sulfate (CS)	[154]
Asialoglycoprotein (ASGP) receptor	Galactose (Gal)	[155]
Glycyrrhetinic acid (GA) receptor	GA	[13]
Integrin receptor αvβ3	RGD peptide	[143]
Glucose transporters	Glucose dervative	[76]
Membrane internalization receptors (LRP1 and LRP2) and Tf receptor	Lactoferrin (Lf)	[156]
Amino acids transporters	Lysine	[157]
Carbonic anhydrase IX (CA IX)	Anti-carbonic anhydrase IX (CA IX) antibody	[158]
Nucleolin	Nucleolin-specific aptamer (AS1411)	[159]
Scavenger receptor B	High-density lipoprotein (HDL)	[160]

**Table 4:** Ligands mediated drug delivery systems of natural medicines.

Ligand	Drug Delivery System	Natural Products	Cancer Type	In Vitro and In Vivo Evaluations	Ref.
FA	FA-BAS NPS/BA	Baicalin	Breast cancer	(1) Increasing targeted uptake efficiency and cytotoxicity; (2) Promoting apoptosis by increasing the expression of caspase-8 and reactive oxygen species (ROS) and decreasing the level of Bid.	[161]
	PLGA-PEG-FA NPs	Genistein	Ovarian cancer	(1) Exhibiting sustained release of drug for around six days; (2) Increasing cellular uptake.	[162]
	FA-Cur-NPs	Curcumin	Cervical cancer	(1) Showing superior cytotoxic activity and drug uptake through FR-mediated endocytosis pathways; (2) Distributing mainly in in tumor sites of Hela xenograft mouse model and significantly inhibiting tumor growth; (3) Possessing a high safety that there was no significant change in body weight of mouse after 30 days of treatment.	[152]
	Cur/FA-PEG-PLA	Curcumin	Glioblastoma multiforme	(1) Suppressing the growth of GL261 cells and promoting apoptotic rate; (2) The tumor growth of tumor-bearing mice processed with Cur/FA-PEG-PLA were repressed via suppressing angiogenesis and facilitating apoptosis.	[163]
	FA-MPEG-PCL/CUR	Curcumin	Colorectal cancer	(1) Increasing the t_1/2_ and AUC; (2) Showing the most significant inhibitory and apoptotic effects on cell growth; (3) Having a stronger effect on inhibiting tumor growth, promoting tumor apoptosis, and weakening tumor angiogenesis than free Cur and unmodified micelles.	[164]
	FA-F108	Evodiamine	Cervical cancer	(1) Showing dose-dependent and time-dependent cytotoxicity against Hela cells; (2) Greatly inducing apoptosis as compared to pure drug; (3) Improving intracellular delivery of evodiamine through overexpressed folate receptors.	[165]
	5-FAM/FA/TP@Fe-MIL-101	Triptolide	Liver cancer	(1) Showing better targeted therapy efficiency and reducing the systemic toxicity of triptolide; (2) The modification of 5-FAM facilitated fluorescence imaging of the tumor site and realized the construction of an integrated nano-platform for fluorescence imaging and treatment.	[166]
	C-RSV-FER-FA-SLNs	Resveratrol and ferulic acid	Colon cancer	Compared with free drugs, FA-modified formulations increased the cytotoxicity of cancer cells, thereby inducing cell apoptosis.	[167]
	RSV-FA-NLCs	Resveratrol	Breast cancer	(1) Compared with unmodified NLCs, folate-modified NLCs exhibited higher cytotoxicity on MCF-7 cells overexpressing folate receptors at high levels; (2) The AUC value of RSV-FA-NLCs increased by 9 times compared to free drug (57.92 ± 4.15, 6.37 ± 1.16 (μg/mL)·h, respectively).	[14]
Tf	Tf-PIP-NPs	Piperine	Liver cancer and breast cancer	(1) Compared with unmodified preparations, Tf-PIP-NPs had strong cytotoxicity; (2) Tf-PIP-NPs could reduce mitochondrial membrane potential and induce apoptosis through mitochondrial pathway.	[129]
	Tf-LipoMof@PL	Piperlongumine	Breast cancer	The modification of Tf enhanced the endocytosis of cells towards the formulation, thereby strengthening the ferroptotic cell death.	[168]
	MSN-NH2-Cur-PEG-Tf	Curcumin	Pancreatic cancer	(1) The uptake of the formulation by tumor cells was 7 times higher than that of free drugs; (2) The cytotoxicity of the preparation was 3 times higher than that of free curcumin; (3) MSN-NH2-Cur-PEG-Tf could inhibit tumor growth and reduce tumor metastasis to non-tumor sites.	[169]
	Tf-functionalised microemulsion (Tf-EC-MEs)	ꞵ-Elemene and celastrol	Lung cancer	(1) Improving cell uptake of A549, exhibiting stronger anti-proliferative effects and higher cell apoptosis rates; (2) In the xenograft mouse tumor model carrying A549, Tf-EC-MEs showed enhanced anti-tumor activity compared to all other treatments and did not cause significant systemic toxicity.	[170]
	Tf-LP-CA	Carnosic acid	Liver cancer	(1) Inducing higher levels of apoptosis and reducing the mitochondrial membrane potential more effectively in HepG2- and SMMC-7721 cells; (2) Regulating the expressions of cleaved poly(ADP-ribose) polymerase, caspase-3 and -9, and B-cell lymphoma 2 (Bcl2) family members.	[128]
HA	dHAD-QT	Quercetin	Breast cancer	(1) Compared with unmodified formulations, the CD44 targeting ability of dHAD-QT micelles was significantly improved; (2) Exhibiting high cytotoxicity and the ability to induce cell apoptosis; (3) Effectively inhibiting tumor growth in tumor-bearing mice, with a tumor inhibition rate of 91.8%.	[134]
	HA-KA-NLCs	Kaempferol	Non-small-cell lung cancer	(1) Inhibiting the proliferation, migration and invasion of A549 cells, promoting cell apoptosis and increasing cell uptake; (2) Activating the epithelial–mesenchymal transition (EMT)-related signaling pathway and regulating the expression of E-cadherin, N-cadherin, and Vimentin in A549 cells.	[136]
	HA-mPEG-CTD-NLC	Cantharidin	Liver cancer	(1) The t_1/2_, AUC, and plasma clearance rates of the formulation were higher than those of the cantharidin solution; (2) Having superior cytotoxicity and targeting effects on SMMC-7721 cells; (3) Significantly inhibiting tumor growth and prolonged survival in tumor-bearing mice, with a tumor inhibition rate of 65.96%.	[171]
	Cur-HA NPs	Curcumin	Colon cancer	(1) Nanoparticles can effectively orally deliver drugs to the lower gastrointestinal tract to treat local colon diseases; (2) The modification of HA can effectively increase the uptake of the formulation by HT-29 cells.	[137]
	UA/(AS-IV)@PDA-HA	Ursolic acid and astragaloside IV	Non-small-cell lung cancer	UA/(AS-IV)@PDA-HA could be used for chemotherapy, photothermal therapy, and immunotherapy simultaneously. In this nanosystem, HA could improve drug targeting, ursolic acid exerted cytotoxic effects, astragaloside IV mediated autoimmune response, and polydopamine-mediated photothermal therapy inhibits tumor growth.	[172]
	HA-DOPE@Lips/HNK	Honokiol	Osteosarcoma	(1) HA-DOPE@Lips/HNK could inhibit cell proliferation, cause apoptosis, arrest the cell cycle in the G1 phase, and disrupt mitochondrial activity. (2) In vivo experiments indicated that HA-DOPE@Lips/HNK specially delivered the drug into the tumor and inhibited tumor growth and showed no evident toxicity to normal tissues.	[173]
Gal	Gal-SP188–PLGA	Resibufogenin	Liver cancer	Both cellular and animal experiments had shown that the preparation had strong liver targeting properties.	[139]
	API-GAL-NPs	Apigenin	Liver cancer	(1) API-GAL-NPs could better improve the intracellular internalization of drugs, thereby significantly increasing the cytotoxicity and apoptotic potential of HepG2 cells; (2) By the significant reduction in nodule formation, downregulation of matrix metalloproteinases (MMP-2 and MMP-9), and induction of apoptosis in the liver, API-GAL-NPs had a better protective effect on DEN-induced liver cancer in rats.	[174]
	Microemulsion (Gal(oct)-C-ME)	Coix seed oil, coixan	Liver cancer	(1) The internalized Gal(oct)-C-ME was 2.28 times higher than that of formulations without Gal modification; (2) Tumor-bearing mice were gavaged with Gal(oct)-C-ME for 14 days, which had the strongest inhibitory effect on tumor growth and the lowest toxicity to the liver and kidneys.	[155]
	GF68-Gal	Galangin	Liver cancer	(1) Increasing the accumulation of the preparation in the liver; (2) Inhibiting P-pg and cytochrome enzyme, thus inhibiting drug effluence and metabolism	[46]
RGD	HCPT@NMOFs-RGD	10-Hydroxycamptothecin	Liver cancer	(1) HCPT@NMOFs-RGD were specifically enriched in the tumor by binding specifically to integrin αvβ3 and led to a reduction in tumor volume; (2) The xenografts in mice were eliminated remarkably following HCPT@NMOFs-RGD treatment with laser irradiation.	[175]
	RGD-EG-SS-PTX	Paclitaxel	Gastric cancer	(1) This micelle had a controlled release function and could decompose and ultimately release PTX under the reduction in glutathione (GSH) in tumor cells; (2) It could target gastric cancer cells and inhibit cell proliferation by inducing apoptosis; (3) In vivo experiments had shown that PTX micelles could be effectively delivered to the tumor site and inhibit tumor growth.	[144]
	RGD modified liposomes (phosphatidylcholine/cholesterol/DSPE-PEG2000-RGD)	Vinorelbine and tetrandrine	Brain glioma	It could significantly enhance the transport between brain barriers, accumulate significantly in glioma cells, and have a significant inhibitory effect on mouse glioma.	[143]
	RGD-Lip-Cur	Curcumin	Breast cancer	Promoting apoptosis by activating caspase 3/7.	[145]
	RGD-HSA-GEM/CUR NPs	Gemcitabine and curcumin	Pancreatic cancer	In vivo research indicated that RGD-HSA-GEM/CUR NPs had significant targeting effects on subcutaneous tumors.	[176]

Notes: FA represents folate. Tf represents transferrin. HA represents hyaluronic acid. Gal represents galactose. RGD represents RGD peptide. AUC and t_1/2_ mean area under the curve and elimination half-life, respectively.

**Table 5:** Some multi-layer nano-preparations of natural medicines.

Drug	Targeting Ligands	Cancer Type	Study Interest	Ref.
Quercetin (Que) and paclitaxel (PTX)	NGR and RGD	Breast cancer	It was a matrix metalloproteinase-triggered dual-targeting hybrid micelle-in-liposome system. Que was delivered to tumor tissue under the guidance of NGR to exert anti-fibrotic effects, whereas PTX was delivered to tumor cells as a chemotherapy agent.	[115]
PTX	HA and TPP	Lung cancer	The modification of HA enabled micelles to target tumor cells through CD44 receptors. TPP promoted the accumulation of micelles in mitochondria, which was beneficial for enhancing the anti-tumor effect of PTX and reversing the multiple drug resistance.	[186]
PTX	HA and TPP	Breast cancer	HA and TPP targeted cell membranes and mitochondria, respectively, and boric acid had pH and photothermal responsiveness. This preparation could respond to infrared signals and regulate the release of PTX, which had great potential in tumor imaging and chemical photothermal therapy.	[187]
PTX	FA and BBR	Brain glioma	Liposomes modified by FA could be effectively targeted to glioma cells. BBR could be attracted by mitochondrial membrane potential and concentrate on mitochondria to achieve mitochondrial targeting and induce cell apoptosis.	[215]

Notes: NGR represents NGR peptide. RGD represents RGD peptide. HA represents hyaluronic acid. TPP means triphenylphosphine. FA represents folate. BBR means berberine. CD44 means cluster of differentiation-44.

## Data Availability

Not applicable.
